# Supplementary material for: Asymmetric peptidoglycan editing generates cell curvature in Bdellovibrio predatory bacteria
Source: Nat Commun. 2022 Mar 21;13:1509. doi: 10.1038/s41467-022-29007-y (PMC8938487; doi:10.1038/s41467-022-29007-y)
Supplement: Supplementary file 1 — Supplementary information [file 41467_2022_29007_MOESM1_ESM.pdf]

Supplementary information

Asymmetric peptidoglycan editing generates cell curvature in *Bdellovibrio* predatory bacteria

Banks *et al*.

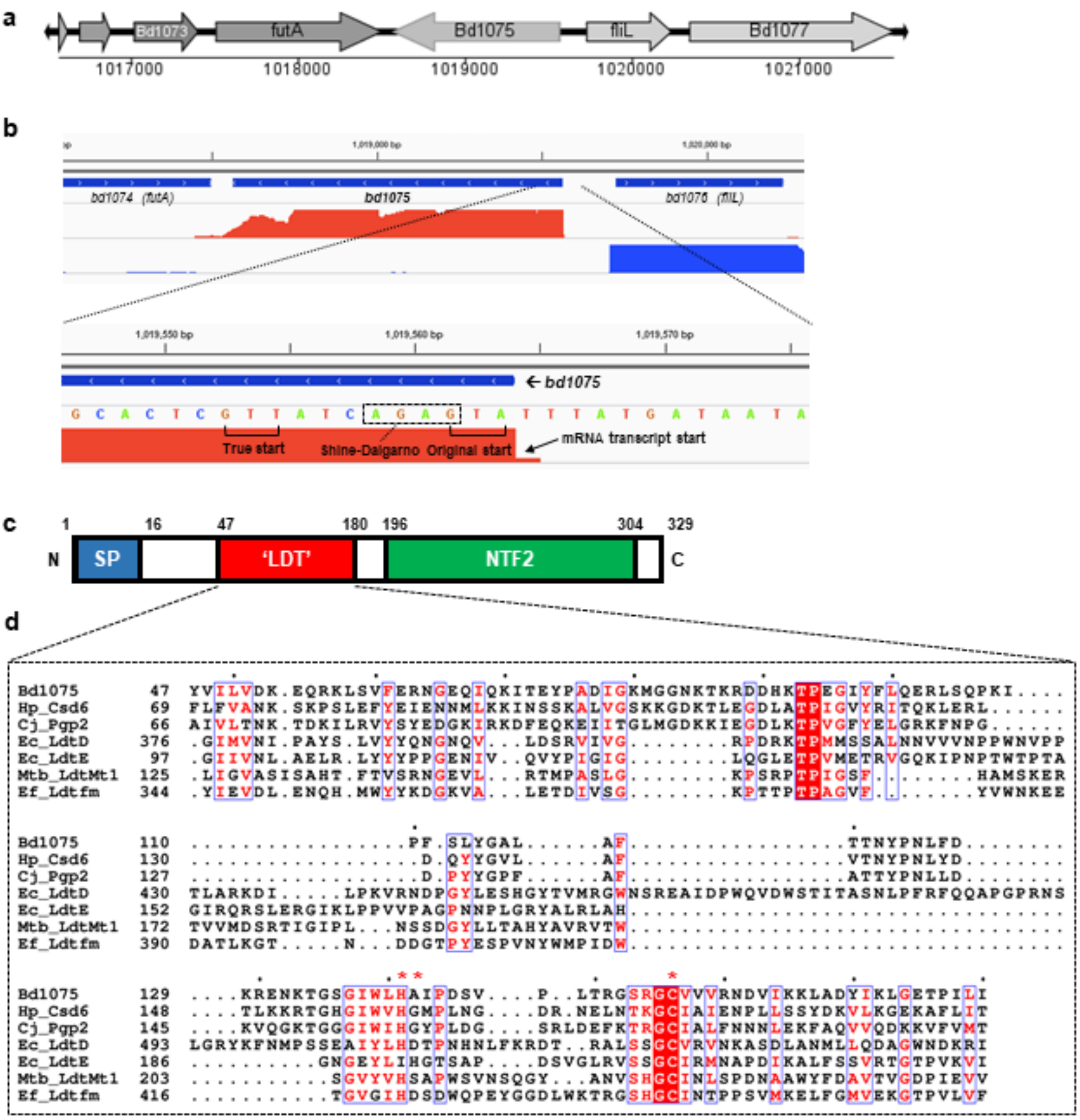

Supplementary Fig. 1 Bioinformatic predictions of Bd1075.

a Genomic location of *bd1075* in the *B. bacteriovorus* HD100 genome, viewed in xBASE[1]. The *bd1075* gene is monocistronic and flanked by divergently transcribed genes. **b** RNA-Seq reads[2] from *B. bacteriovorus* strain HID13 aligned to the *B. bacteriovorus* HD100 genome with Rockhopper[3, 4] and visualized in Integrated Genomics Viewer[5]. The DNA sequence has been reverse-complemented for ease of viewing. The mRNA transcript of *bd1075* begins at the start codon, indicating that the start codon of the gene was probably mis-annotated. The probable Shine-Dalgarno sequence (GAGA) and true start codon (TTG) are annotated. Correcting the start site annotation removes the first 3 residues (MRL) of Bd1075 but does not affect any signal peptide or domain

predictions. **c** Schematic of the predicted domain structure of Bd1075. Numbers indicate residue position; SP: signal peptide; 'LDT': predicted LD-transpeptidase-family domain; NTF2: nuclear transport factor 2-like domain. **d** Multiple sequence alignment of the *B. bacteriovorus* HD100 Bd1075 LDT domain against the LDT domains (in descending order) of: *Helicobacter pylori* Csd6, *Campylobacter jejuni* Pgp2, *Escherichia coli* LdtD, *Escherichia coli* LdtE, *Mycobacterium tuberculosis* Ldt_{Mt1}, and *Enterococcus faecium* Ldt_{fm}. Catalytic triad residues are indicated by red asterisks. The alignment was generated in Clustal Omega[6] and visualized with ESPript 3[7].

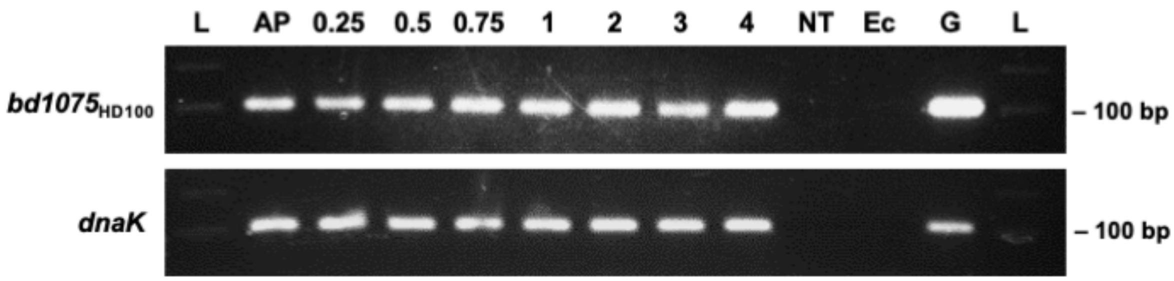

Supplementary Fig. 2 *bd1075*$_{\mathbf{HD100}}$ is constitutively expressed during the predatory cycle. Reverse-transcriptase PCR was performed on *B. bacteriovorus* HD100 RNA isolated at timepoints throughout the predatory cycle using primers designed to amplify a 102 bp product internal to the *bd1075* gene. L: 100 bp NEB molecular weight ladder; AP: attack-phase; 0.25-4: hours since the start of predation; NT: no template RNAase-free water control, Ec: *E. coli* S17-1 RNA control, G: *B. bacteriovorus* HD100 genomic DNA positive control. Both *bd1075* and the control gene *dnaK* are constitutively expressed throughout predation. Two independent biological repeats were carried out. An uncropped gel is provided in the Source Data file.

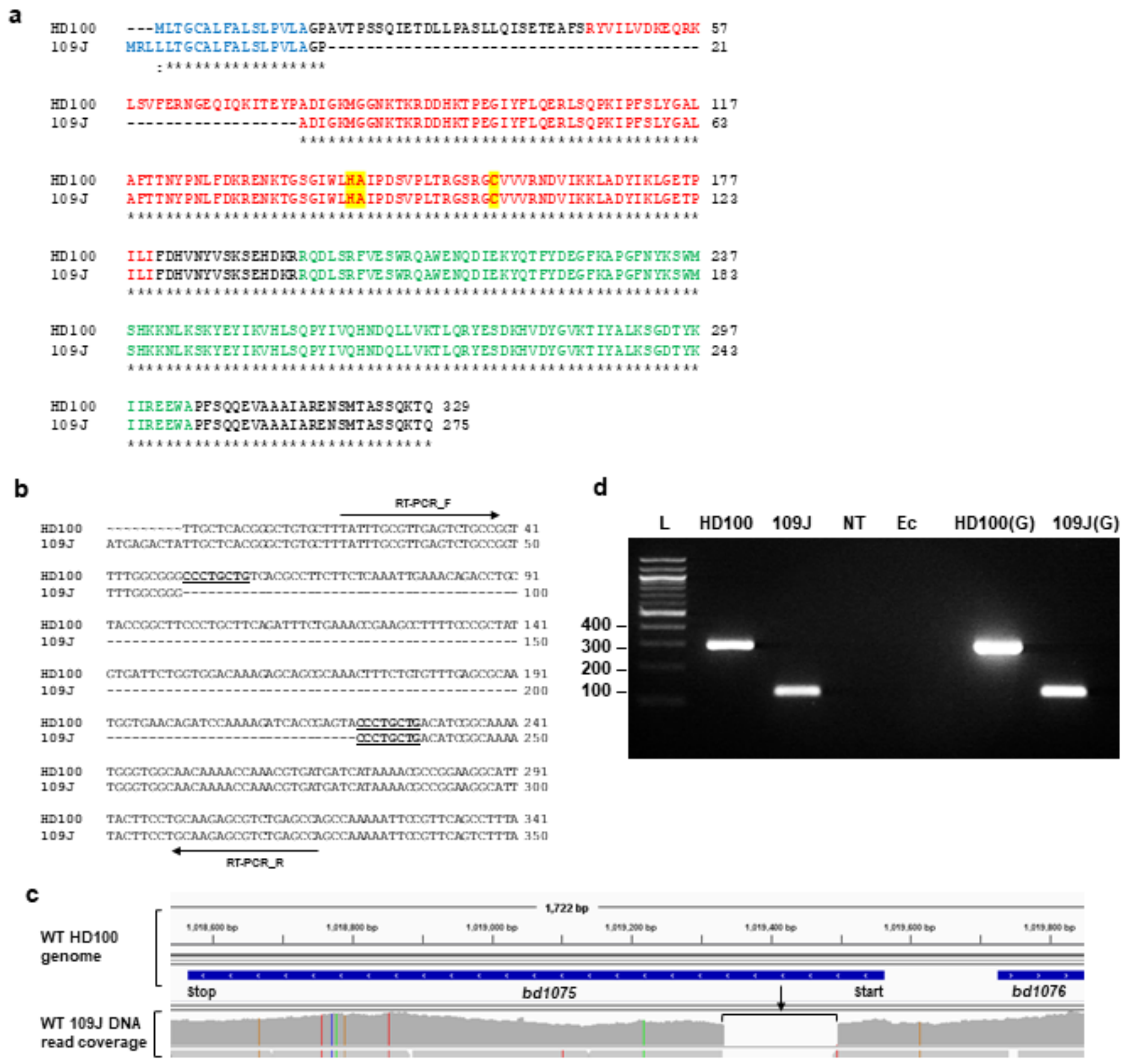

Supplementary Fig. 3 *bd1075* is expressed in strain 109J and contains an N-terminal 57 residue truncation.

a Pairwise amino acid alignment of wild-type $Bd1075_{HD100}$ and wild-type $Bd1075_{109J}$. Asterisks indicate identical protein residues and dashes indicate the 57 amino acid truncation present in $Bd1075_{109J}$ between Proline-18 and Tyrosine-74. Blue residues: signal peptide; red residues: predicted 'LD-transpeptidase (LDT) domain; red residues highlighted in yellow: LDT catalytic triad residues; green residues: NTF2 domain. **b** Pairwise DNA alignment of wild-type HD100 $bd1075_{HD100}$ and wild-type $bd1075_{109J}$ from 1-350 bp, showing the truncation (indicated by dashes) present in strain 109J which is flanked by 8 bp flanking repeats (underlined and emboldened). **c** Wild-type 109J DNA sequence reads[8] mapped to the wild-type HD100 genome, showing the truncation present in $bd1075_{109J}$. Data were visualized in Integrated Genomes Viewer. **d** Reverse-transcriptase PCR was

performed on RNA isolated from attack-phase wild-type HD100 and wild-type 109J using primers (indicated with black arrows in (**b**) designed to anneal to either side of the *bd1075*$_{109J}$ truncation. Expected product sizes of 298 bp (HD100) and 127 bp (109J) confirmed the presence of the 109J truncation in the RNA transcript. L: 100 bp NEB molecular weight ladder; HD100 & 109J: RNA isolated from strains HD100 and 109J; NT: no template RNAase-free water control; Ec: *E. coli* S17-1 RNA control; HD100(G) and 109J(G): *B. bacteriovorus* genomic DNA positive controls. Two independent biological repeats were carried out. An uncropped gel is provided in the Source Data file.

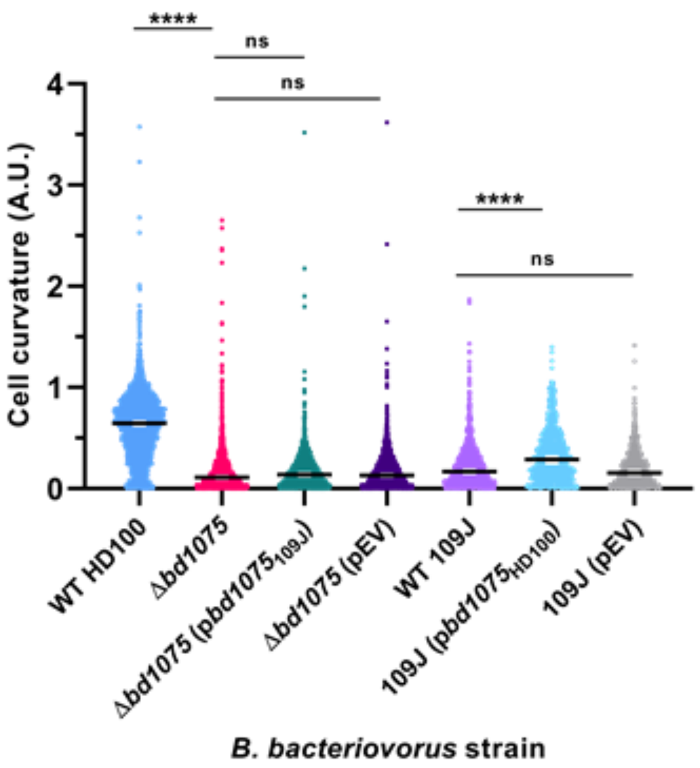

Supplementary Fig. 4 Curvature of *B. bacteriovorus* 109J and additional complementation strains.

Curvature measurements of *B. bacteriovorus* attack-phase cells. n = 2503 cells (WT HD100), 2149 cells (Δ*bd1075*), 1461 cells (Δ*bd1075* (p*bd1075*$_{109J}$)), 2269 cells (Δ*bd1075* (pEV)), 1554 cells (WT 109J), 669 cells (109J (p*bd1075*$_{HD100}$)), or 759 cells (109J (pEV)) per strain from 3 biological repeats. WT HD100, Δ*bd1075* and Δ*bd1075* (pEV) data are reproduced from Fig. 1c. Error bars represent 95% confidence intervals of the median. ns: non-significant (p>0.05), ****: p<0.0001; Kruskal-Wallis test with Dunn's multiple corrections. Frequency distributions are included in Supplementary Fig. 5b. Source Data are provided as a Source Data file.

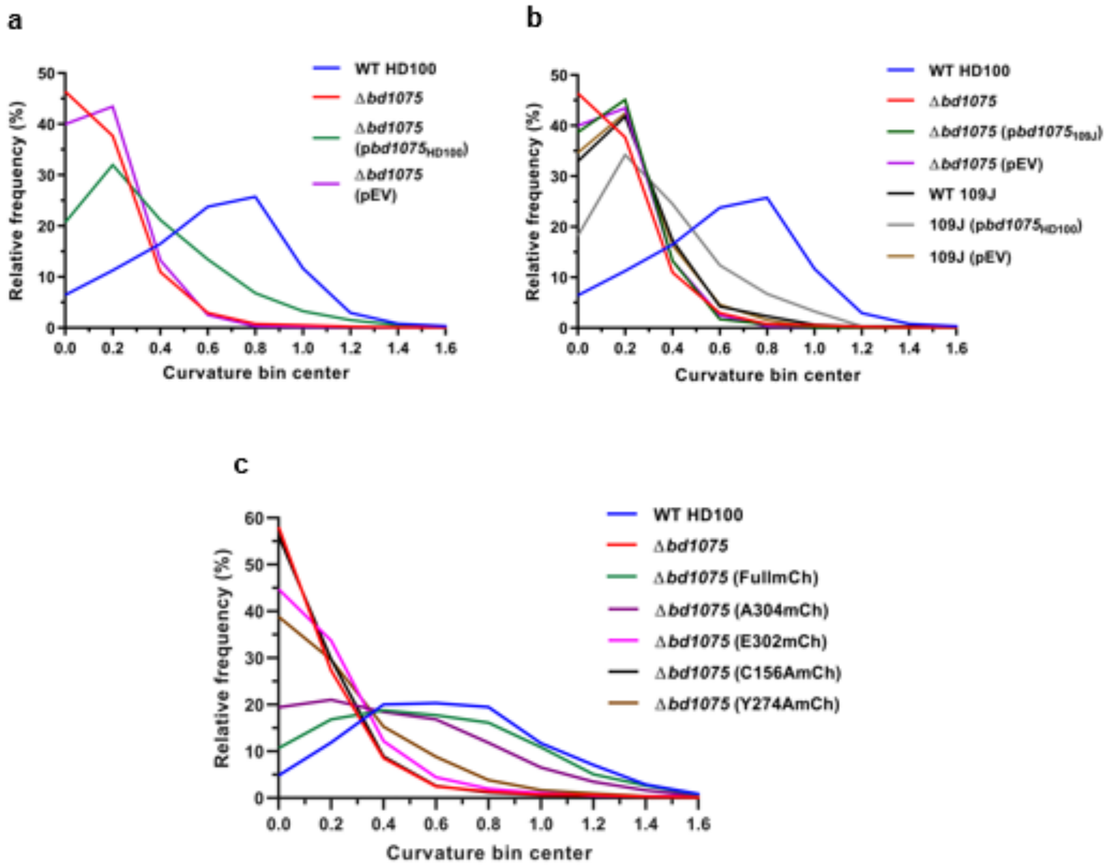

Supplementary Fig. 5 Frequency distributions of *B. bacteriovorus* cell curvature.

Curvature frequency distributions of attack-phase *B. bacteriovorus* strains shown in Fig. 1c (**a**), Supplementary Fig. 4 (**b**) and Fig. 6e (**c**). Graphs show the relative percentage of cells that have a particular value of curvature. In (**a**), n = 2503 cells (WT HD100), 2149 cells (Δ*bd1075*), 1920 cells (Δ*bd1075* (p*bd1075*$_{\text{HD100}}$)), or 2269 cells (Δ*bd1075* (pEV)) per strain from 3 biological repeats. In (**b**), n = 2503 cells (WT HD100), 2149 cells (Δ*bd1075*), 1461 cells (Δ*bd1075* (p*bd1075*$_{\text{109J}}$)), 2269 cells (Δ*bd1075* (pEV)), 1554 cells (WT 109J), 669 cells (109J (p*bd1075*$_{\text{HD100}}$)), or 759 cells (109J (pEV)) per strain from 3 biological repeats. In (**c**), n = 2099 cells (WT HD100), 1886 cells (Δ*bd1075*), 2577 cells (Δ*bd1075* (FullmCh)), 2170 cells (Δ*bd1075* (A304mCh)), 2812 cells (Δ*bd1075* (E302mCh)), 2083 cells (Δ*bd1075* (C156AmCh)) or 2523 cells (Δ*bd1075* (Y274AmCh)) per strain from 3 biological repeats. Bin width = 0.2.

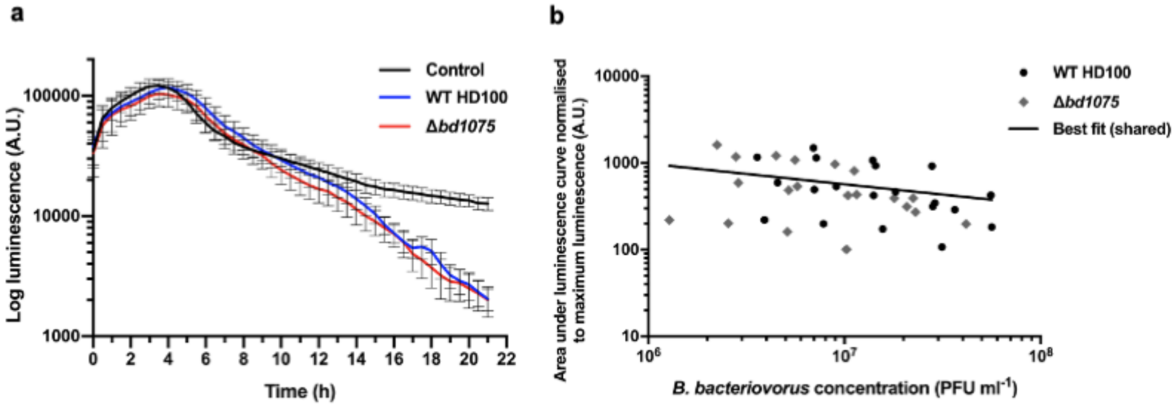

Supplementary Fig. 6 Predation on *E. coli* in liquid culture by *B. bacteriovorus* HD100 wild-type and Δ*bd1075*.

Comparison of the predation efficiency of *B. bacteriovorus* WT HD100 and Δ*bd1075* in liquid culture. Predation efficiency was quantified by the rate in the reduction of *E. coli* S17-1 prey luminescence, measured every 30 min for 21 h. **a** Representative luminescence prey-death curve. Blue line: wild-type HD100; red line: Δ*bd1075*; black line: control of heat-killed predator cells. Error bars represent standard error of the mean. Two-tailed Spearman correlation analyses showed significant (p<0.0001) correlation between Δ*bd1075* and wild-type HD100 revealing no difference between them. **b** The area under each luminescence curve was measured and normalized to the maximum luminescence. These values were plotted against corresponding *B. bacteriovorus* predator concentrations which were enumerated by plaque counts. Black circles: wild-type HD100; grey diamonds: Δ*bd1075*. Both data sets could be analyzed by a shared non-linear regression line of best fit, indicating that there was no significant difference between strains (p=0.70). Data are from 5 biological repeats. Source Data are provided as a Source Data file.

a

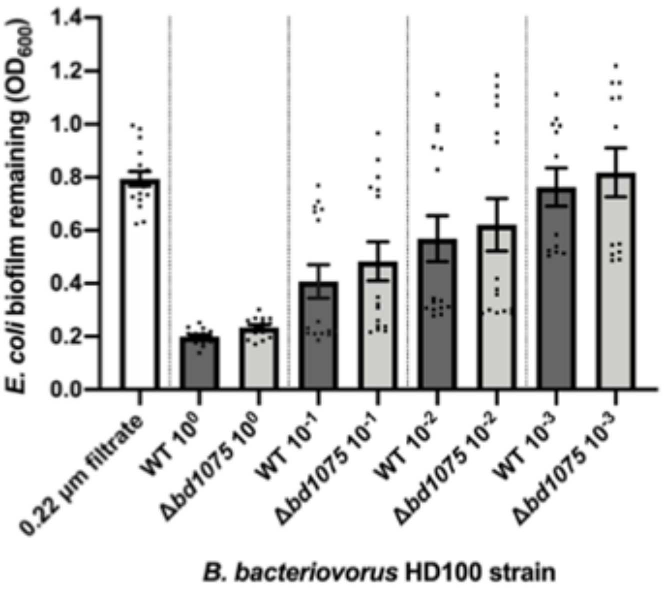

b

Biological repeat	*B. bacteriovorus* count (PFU/ml)	
	Wild-type	Δ*bd1075*
1	5.3e+007	3.8e+007
2	2.05e+007	1.04e+007
3	1.45e+007	8500000

Supplementary Fig. 7 Predation on *E. coli* biofilms by *B. bacteriovorus* HD100 wild-type and Δ*bd1075*.

Comparison of *B. bacteriovorus* WT HD100 and Δ*bd1075* predation upon pre-formed *E. coli* S17-1 biofilms within 96-well PVC microtiter plates. **a** Predation efficiency was assessed by quantification of remaining *E. coli* biofilm (OD_{600}) after 24 h incubation with each predator at a range of dilutions: neat (10^0), 10^{-1}, 10^{-2}, and 10^{-3}. 0.22 μm filtrate: *B. bacteriovorus* filtered through a 0.22 μm membrane to give a no-predator control. Data points represent 15 technical repeats from 3 independent biological repeats and error bars represent the standard error of the mean. There was a small significant difference (p=0.01; unpaired two-tailed t-test) between neat WT and Δ*bd1075*, however the difference was not considered biologically meaningful as it was within the error margins of the experiment; predator plaque enumerations **(b)** showed that the concentration of viable Δ*bd1075* that had been added was slightly lower than the WT. No other comparisons were significant (p>0.05; two-tailed Mann-Whitney test). Source Data are provided as a Source Data file.

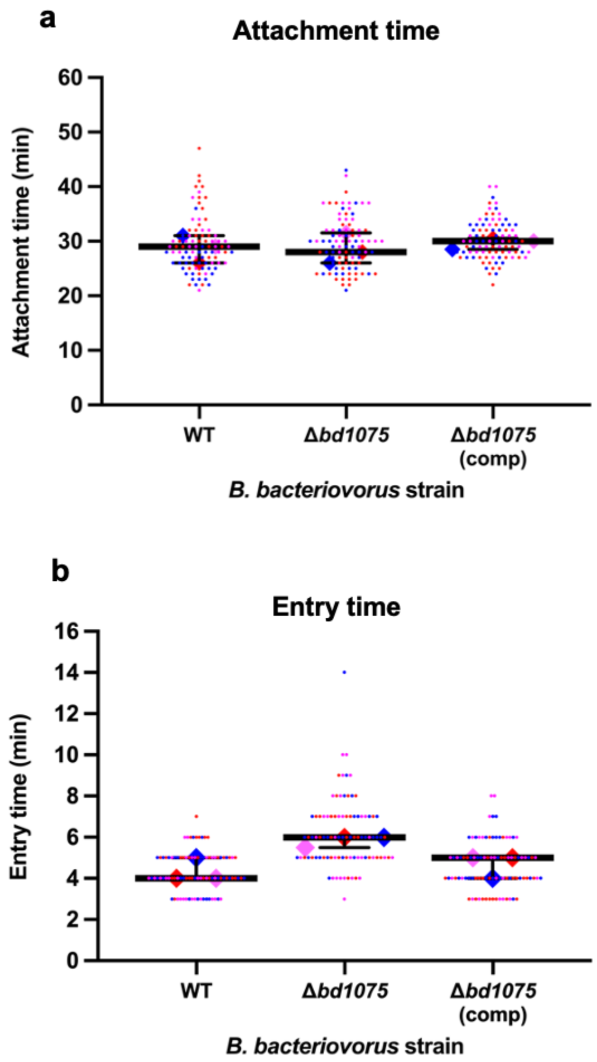

Supplementary Fig. 8. Scatter plots for *B. bacteriovorus* attachment and entry time into *E. coli* prey shown in Fig. 2.

Attachment (**a**) and entry (**b**) times for *B. bacteriovorus* invasion into *E. coli* S17-1 prey presented as scatter super-plots. All data points (90 in total) are shown as bee swarm scatter plots. Data points are whole discrete minutes (e.g., 4 min, 5 min, 6 min), therefore some data points obscure each other on a bee swarm plot. Data points are colored red, blue or pink according to the experimental repeat. The median of each repeat is shown as a larger symbol. Error bars represent 95% confidence intervals of the median. The median of all 3 biological repeats is annotated as a horizontal black line. Attachment times between the 3 strains did not significantly differ ($p>0.05$; Kruskal-Wallis test with Dunn's multiple corrections) but the Δ*bd1075* entry time was significantly higher than both WT and

Δ*bd1075* (comp) ($p<0.0001$; Kruskal-Wallis test with Dunn's multiple corrections). 30 cells were analysed from each of 3 biological repeats. Source Data are provided as a Source Data file.

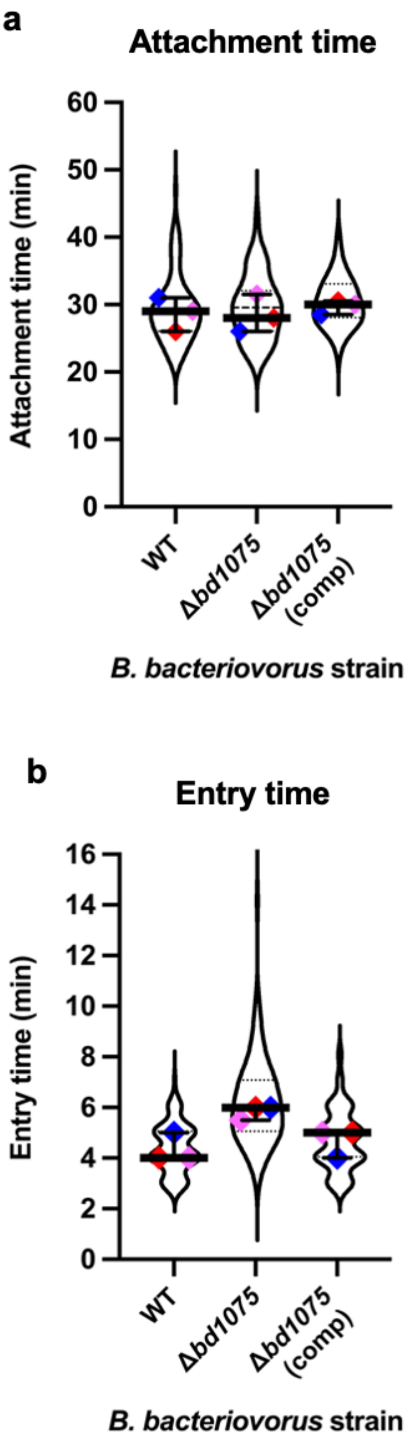

Supplementary Fig. 9. Violin plots for *B. bacteriovorus* attachment and entry time into *E. coli* prey shown in Fig. 2.

Attachment (**a**) and entry (**b**) times for *B. bacteriovorus* invasion into *E. coli* S17-1 prey. All data points (90 in total) are represented as violin plots. The median of each repeat (diamond symbols) is colored red, blue or pink according to the experimental repeat. Error bars represent 95% confidence intervals of the median. The median of all 3 biological repeats is annotated as a horizontal black line.

Dotted lines: upper and lower quartiles. Attachment times between the 3 strains did not significantly differ ($p>0.05$; Kruskal-Wallis test with Dunn's multiple corrections) but the Δ*bd1075* entry time was significantly higher than both WT and Δ*bd1075* (comp) ($p<0.0001$; Kruskal-Wallis test with Dunn's multiple corrections). 30 cells were analysed from each of 3 biological repeats. Source Data are provided as a Source Data file.

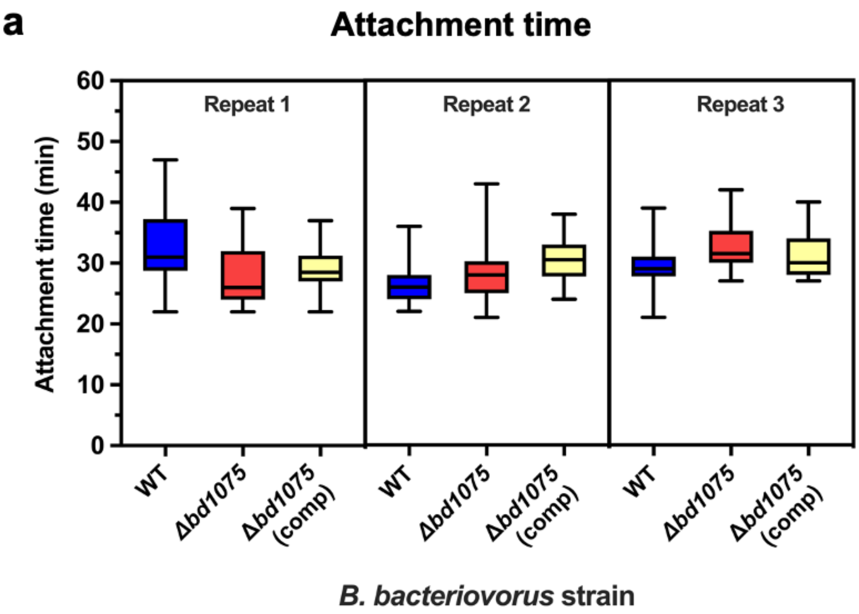

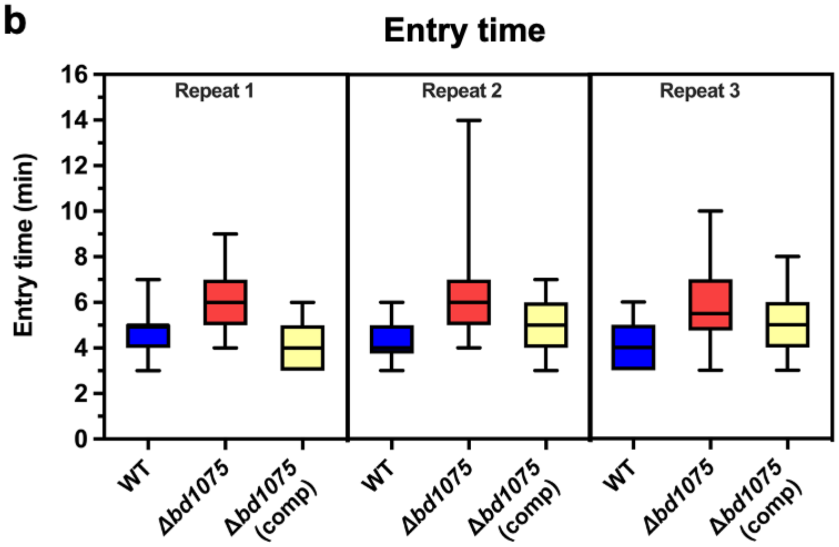

Supplementary Fig. 10. Box-and-whisker plots for each biological repeat of *B. bacteriovorus* attachment and entry time into *E. coli* prey shown in Fig. 2.

Attachment (**a**) and entry (**b**) times for *B. bacteriovorus* invasion into *E. coli* S17-1 prey by wild-type (WT), Δ*bd1075* and Δ*bd1075* (comp) strains, with the full data distribution of each of 3 biological repeats depicted individually as box-and-whisker plots. Box: 25th to 75th percentiles; whiskers: range min-max; box line: median. 30 cells were analysed from each of 3 biological repeats. Source Data are provided as a Source Data file.

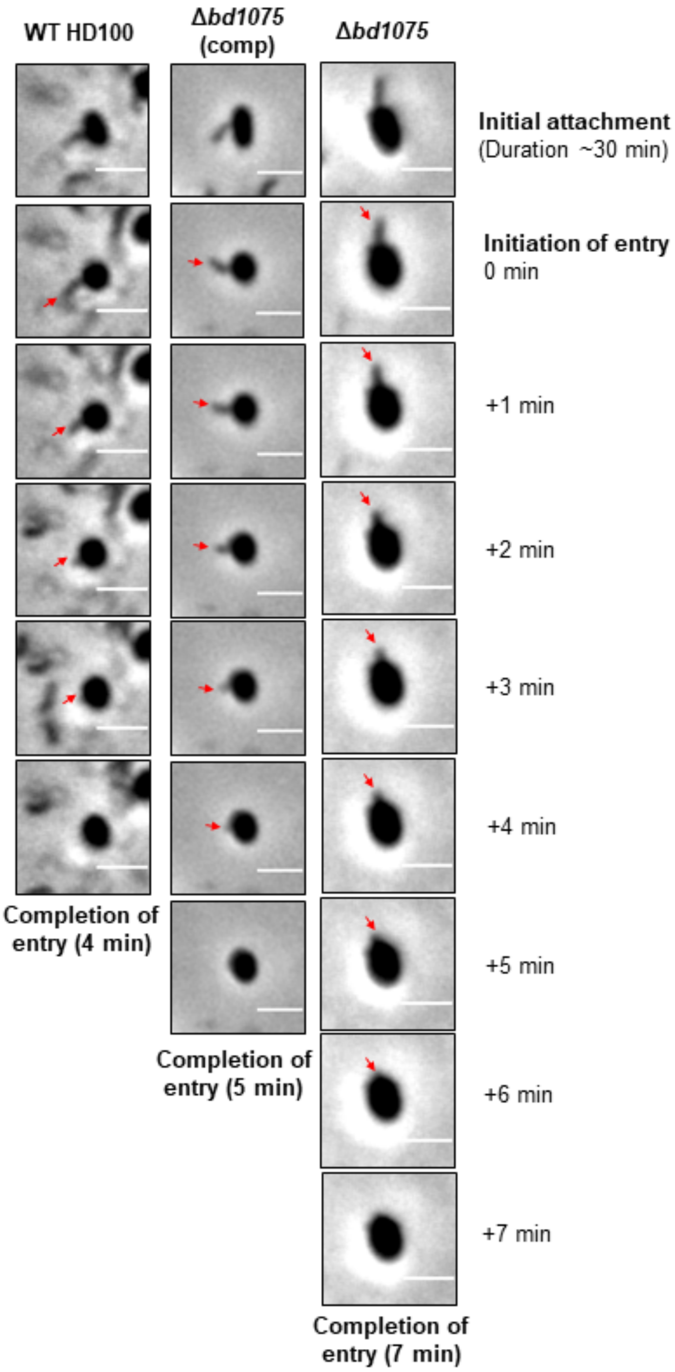

Supplementary Fig. 11 Examples of prey invasion by *B. bacteriovorus* strains

Time-lapse microscopy stills of a typical *B. bacteriovorus* invasion into *E. coli* S17-1 prey by wild-type (WT HD100) *B. bacteriovorus*, *Δbd1075* or complemented *Δbd1075: Δbd1075* (comp). Red arrows indicate the invading *B. bacteriovorus* predator cell from initiation of prey entry until the predator cell has completed entry into prey. Scale bar = 2 μm.

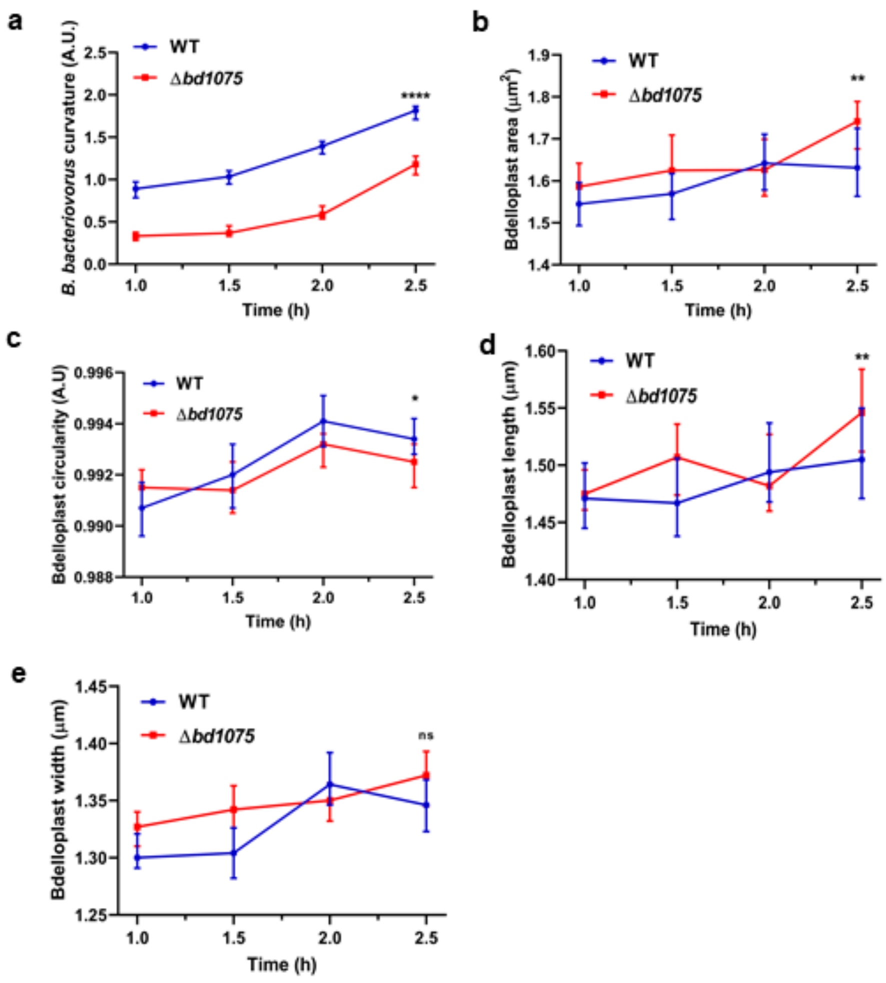

Supplementary Fig. 12 Intra-bacterial growth and bdelloplast topology effects of *B. bacteriovorus* strains: medians

Data from Fig. 3 shown with medians**. a** Curvature of *B. bacteriovorus* WT and Δ*bd1075* strains during predation upon *E. coli* S17-1 pZMR100. n = 134-250 cells per strain and per timepoint from 3 biological repeats. T = hours elapsed since predators and prey were mixed. Error bars represent 95% confidence intervals of the median. ****: p<0.0001; two-tailed Mann-Whitney test. **b** Area, **c** circularity, **d** length and **e** width of *E. coli* prey bdelloplasts during predation by WT or Δ*bd1075* predators. For data in (c), (d), (e), (f) and (g), n = 169 cells (1 h), 134 cells (1.5 h), 150 cells (2 h) and 160 cells (2.5 h) for the wild-type strain and n = 205 cells (1 h), 160 cells (1.5 h), 245 cells (2 h) and 250 cells (2.5 h) for Δ*bd1075* from 3 biological repeats. T = hours elapsed since predators and prey were mixed. Error bars represent 95% confidence intervals of the median. ns: non-significant (p=0.053); **: p=0.0039 (Area) or p=0.0083 (Length), *: p=0.031; two-tailed Mann-Whitney test. Source Data are provided as a Source Data file.

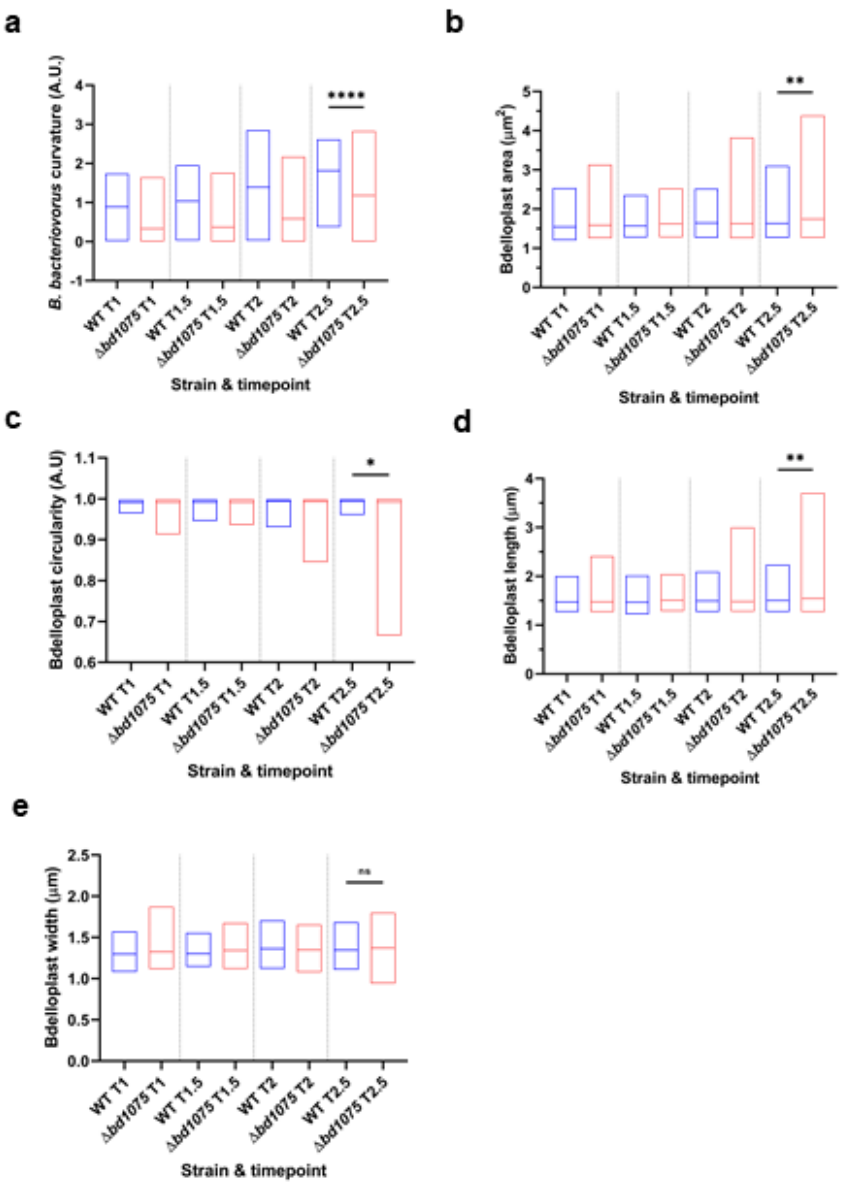

Supplementary Fig. 13 Intra-bacterial growth and bdelloplast topology effects of *B. bacteriovorus* strains: full data distribution

Full data distribution plots for Fig. 3. **a** Curvature of *B. bacteriovorus* WT and Δ*bd1075* strains during predation upon *E. coli* S17-1 pZMR100. n = 134-250 cells per strain and per timepoint from 3 biological repeats. T = hours elapsed since predators and prey were mixed. ****: $p<0.0001$; two-tailed Mann-Whitney test. **b** Area, **c** circularity, **d** length and **e** width of *E. coli* prey bdelloplasts during predation by WT or Δ*bd1075* predators. For data in (c), (d), (e), (f) and (g), n = 169 cells (1 h), 134 cells (1.5 h), 150 cells (2 h) and 160 cells (2.5 h) for the wild-type strain and n = 205 cells (1 h), 160 cells (1.5 h), 245 cells (2 h) and 250 cells (2.5 h) for Δ*bd1075* from 3 biological repeats. T = hours

elapsed since predators and prey were mixed. ns: non-significant ($p=0.053$); **: $p=0.0039$ (Area) or $p=0.0083$ (Length), *: $p=0.031$; two-tailed Mann-Whitney test. Source Data are provided as a Source Data file.

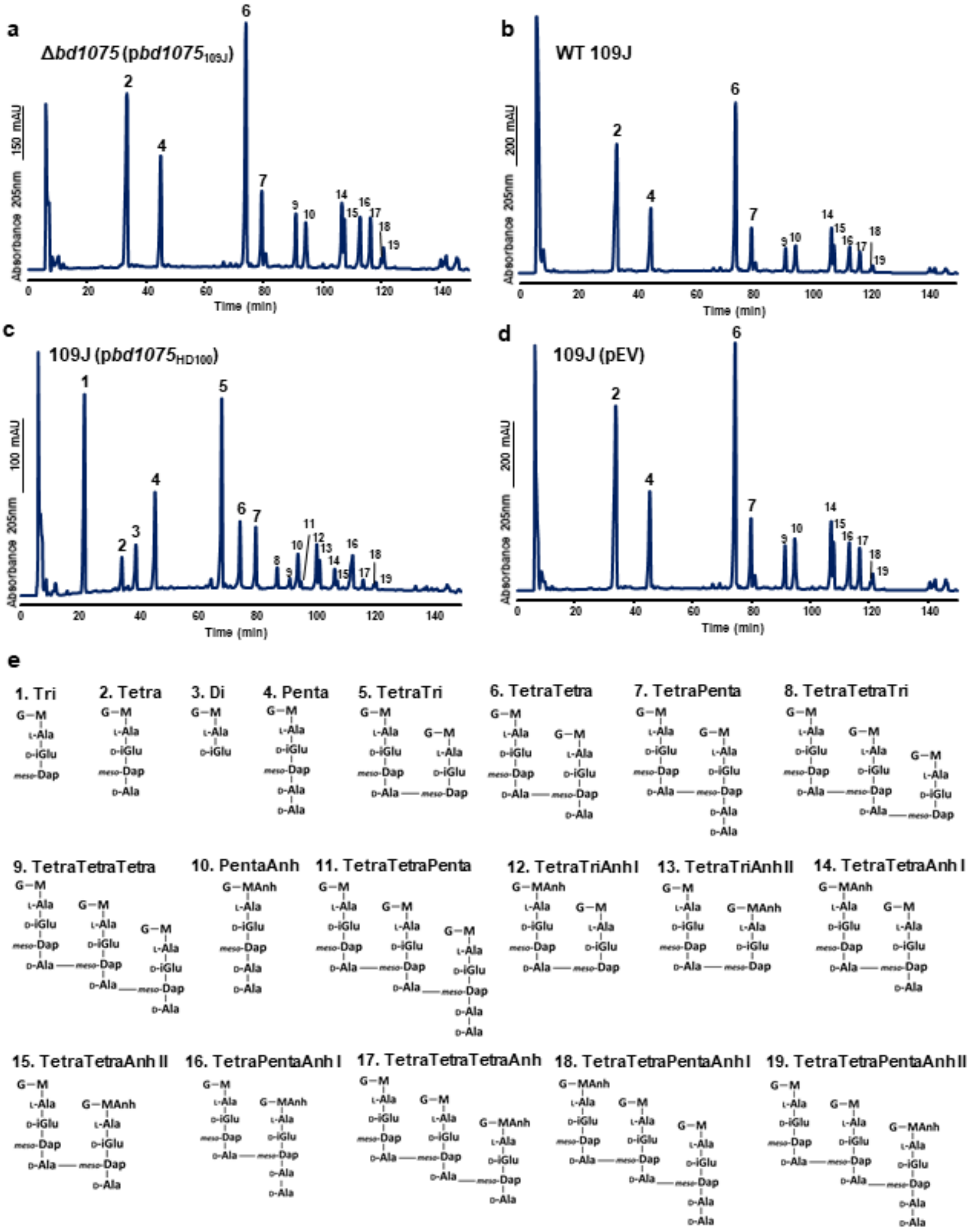

Supplementary Fig. 14 Muropeptide analysis of *B. bacteriovorus* HD100 and 109J strains.

a-d Muropeptide elution profiles obtained by HPLC. Peptidoglycan sacculi were isolated from attack-phase *B. bacteriovorus* cells of **a** HD100 Δ*bd1075* (p*bd1075*$_{109J}$) - *bd1075*$_{109J}$ expressed in Δ*bd1075*, **b** wild-type (WT) 109J, **c** 109J (p*bd1075*$_{HD100}$) - *bd1075*$_{HD100}$ expressed in 109J, and **d** 109J (pEV) - empty vector (EV) control in 109J. Sacculi were digested by cellosyl and the resulting muropeptides

were reduced with sodium borohydride and analyzed by HPLC. Representative chromatograms of 2 biological repeats are shown. **e** Proposed structures of muropeptides. Numbers correspond to those above peaks in **a-d** and were either assigned based on known elution times of the corresponding *E. coli* muropeptides (peaks 1-7) or by mass spectrometry (peaks 8-19) (Supplementary Table 2). G: *N*-acetylglucosamine, M: *N*-acetylmuramitol, MAnH: 1,6-anhydro-*N*-acetylmuramic acid, L-Ala: L-alanine, D-iGlu: D-glutamic acid, *meso*-Dap: *meso*-diaminopimelic acid, D-Ala: D-alanine.

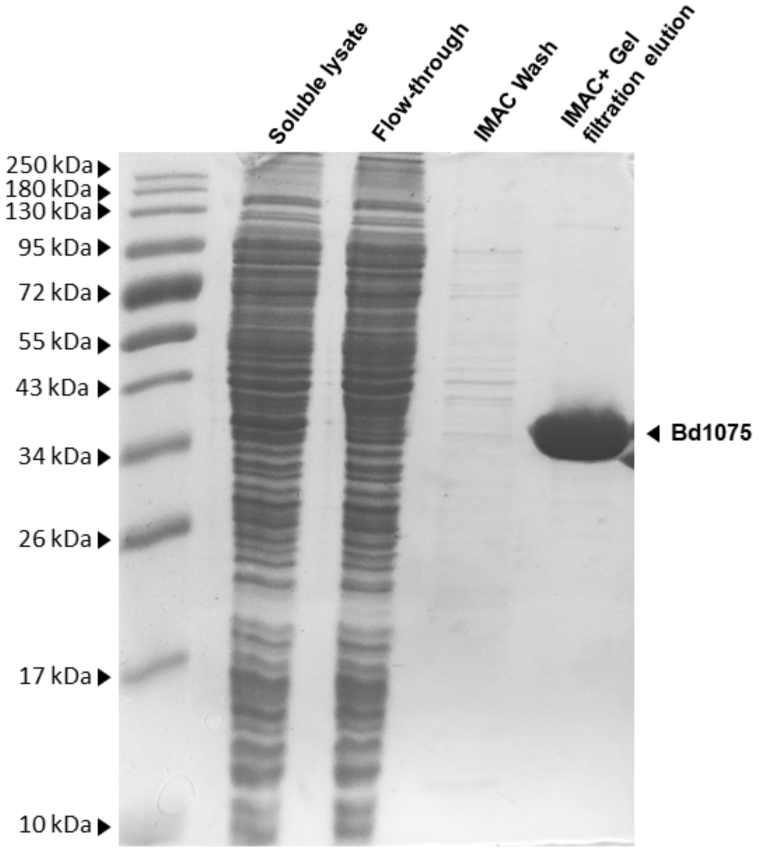

Supplementary Fig. 15 Purification of recombinant Bd1075 protein

Recombinant Bd1075 (35 kDa) was purified by IMAC Ni-NTA and gel filtration chromatography. The purity of the obtained sample was evaluated by 15% SDS-PAGE with Coomassie blue stain. The gel image is representative of 3 independent repeats.

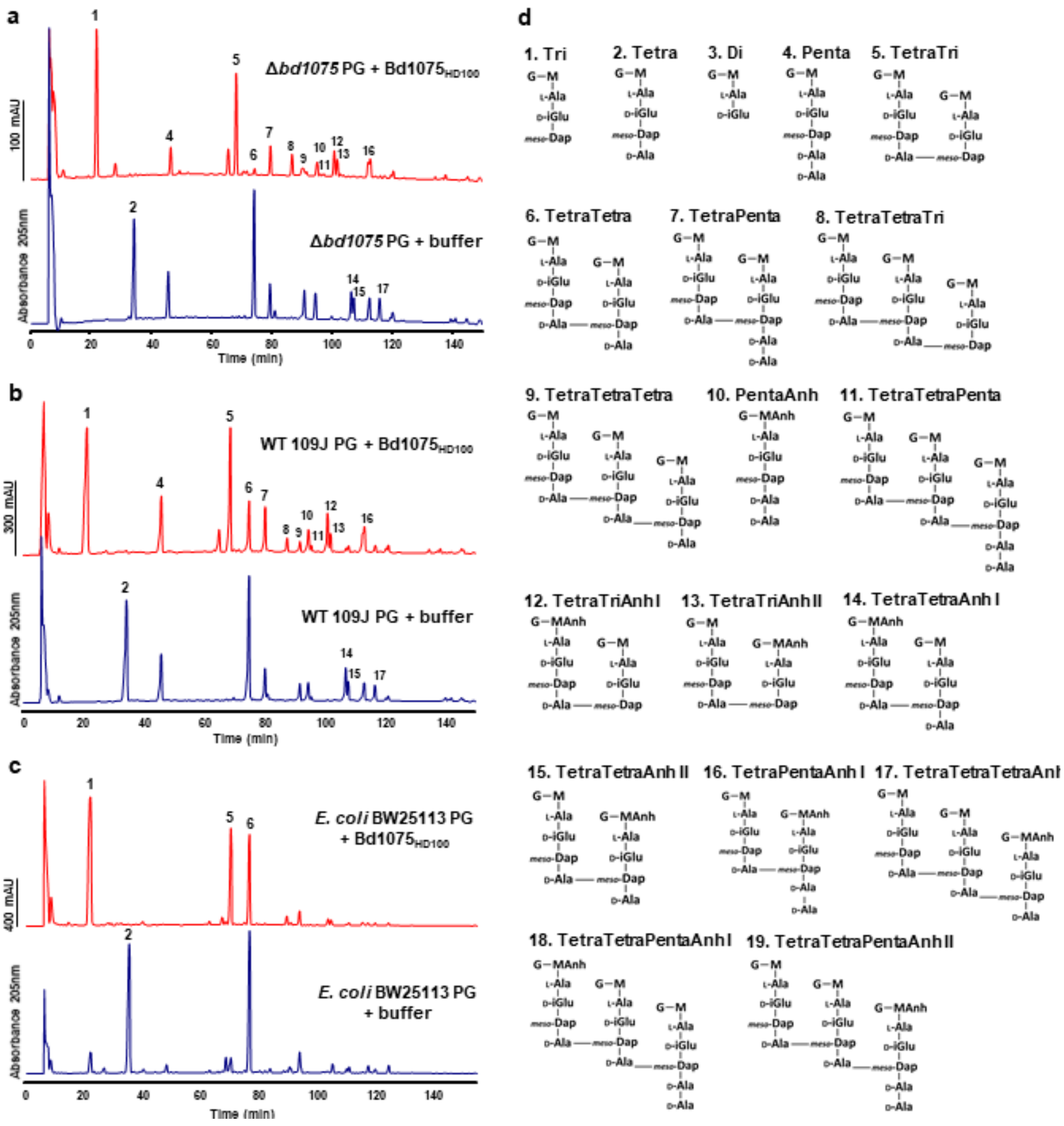

Supplementary Fig. 16 Muropeptide analysis of peptidoglycan sacculi treated with purified Bd1075 *in vitro*.

HPLC elution profiles of muropeptides released from peptidoglycan sacculi from **a** *B. bacteriovorus* Δ*bd1075*, **b** *B. bacteriovorus* wild-type 109J, and **c** *E. coli* wild-type BW25113 that had been treated with either purified Bd1075$_{HD100}$ enzyme (top) or buffer control (bottom). Data are from 1 biological repeat. **d** Proposed structures of muropeptides. Numbers correspond to those above peaks in **a-c** and were either assigned based on known elution times of the corresponding *E. coli* muropeptides (peaks 1-7) or by mass spectrometry (peaks 8-19) (Supplementary Table 2). G: *N*-

acetylglucosamine, M: *N*-acetylmuramitol, MAnh: 1,6-anhydro-*N*-acetylmuramic acid, L-Ala: L-alanine, D-iGlu: D-glutamic acid, *meso*-Dap: *meso*-diaminopimelic acid, D-Ala: D-alanine.

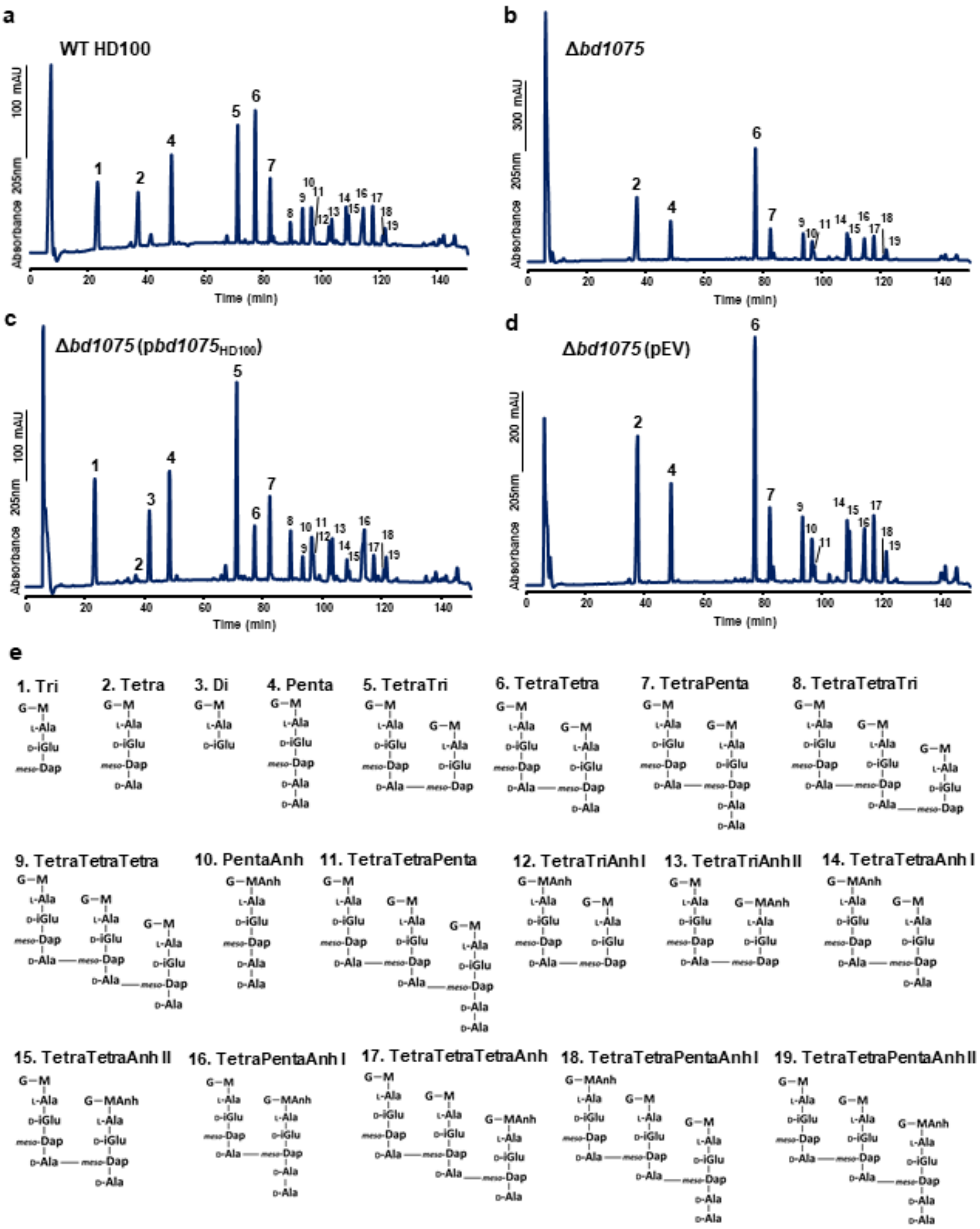

Supplementary Fig. 17 Muropeptide analysis of *B. bacteriovorus* HD100.

a-d Muropeptide elution profiles obtained by HPLC. Peptidoglycan sacculi were isolated from attack-phase *B. bacteriovorus* cells of **a** wild-type (WT) HD100, **b** Δ*bd1075*, **c** Δ*bd1075* (p*bd1075*$_{HD100}$) - *bd1075*$_{HD100}$ expressed in Δ*bd1075*, and **d** Δ*bd1075* (pEV) - empty vector control in Δ*bd1075*. . Sacculi were digested by cellosyl and the resulting muropeptides were reduced with sodium

borohydride and analyzed by HPLC. Representative chromatograms of 2 biological repeats are shown. **e** Proposed structures of muropeptides. Numbers correspond to those above peaks in **a-d** and were either assigned based on known elution times of the corresponding *E. coli* muropeptides (peaks 1-7) or by mass spectrometry (peaks 8-19) (Supplementary Table 2). G: *N*-acetylglucosamine, M: N-acetylmuramitol, MAnh: 1,6-anhydro-*N*-acetylmuramic acid, L-Ala: L-alanine, D-iGlu: D-glutamic acid, *meso*-Dap: *meso*-diaminopimelic acid, D-Ala: D-alanine.

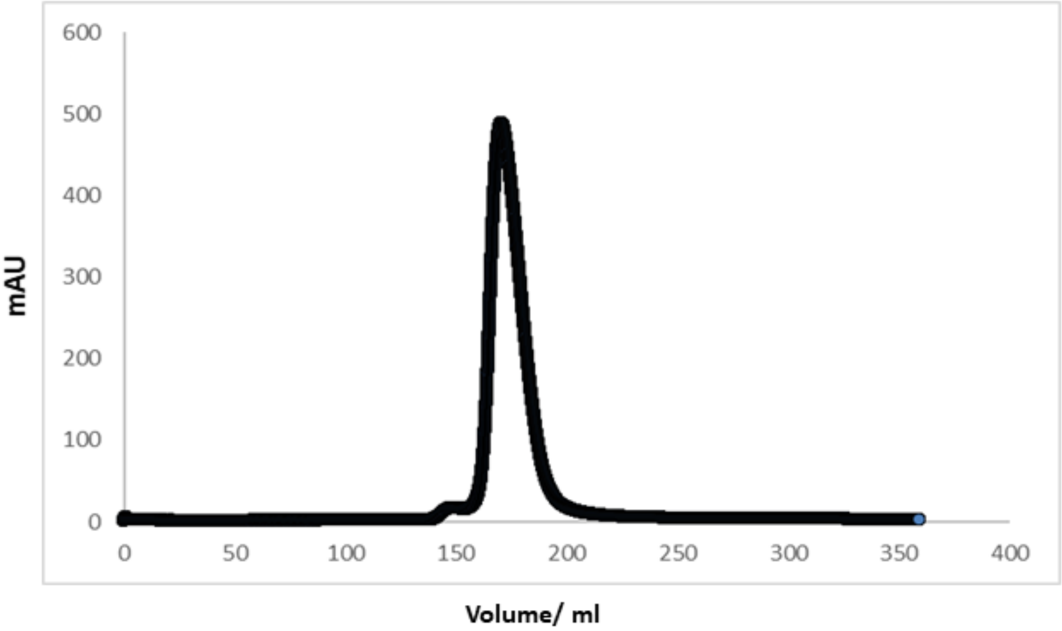

Supplementary Fig. 18 Gel filtration profile of Bd1075 protein

Bd1075 gel filtration profile on a Superdex 75 26/60 column. The profile depicts a monodisperse peak at 170 ml corresponding to monomeric Bd1075. The monomeric peak of Bd1075 was pooled for crystallization and structure determination.

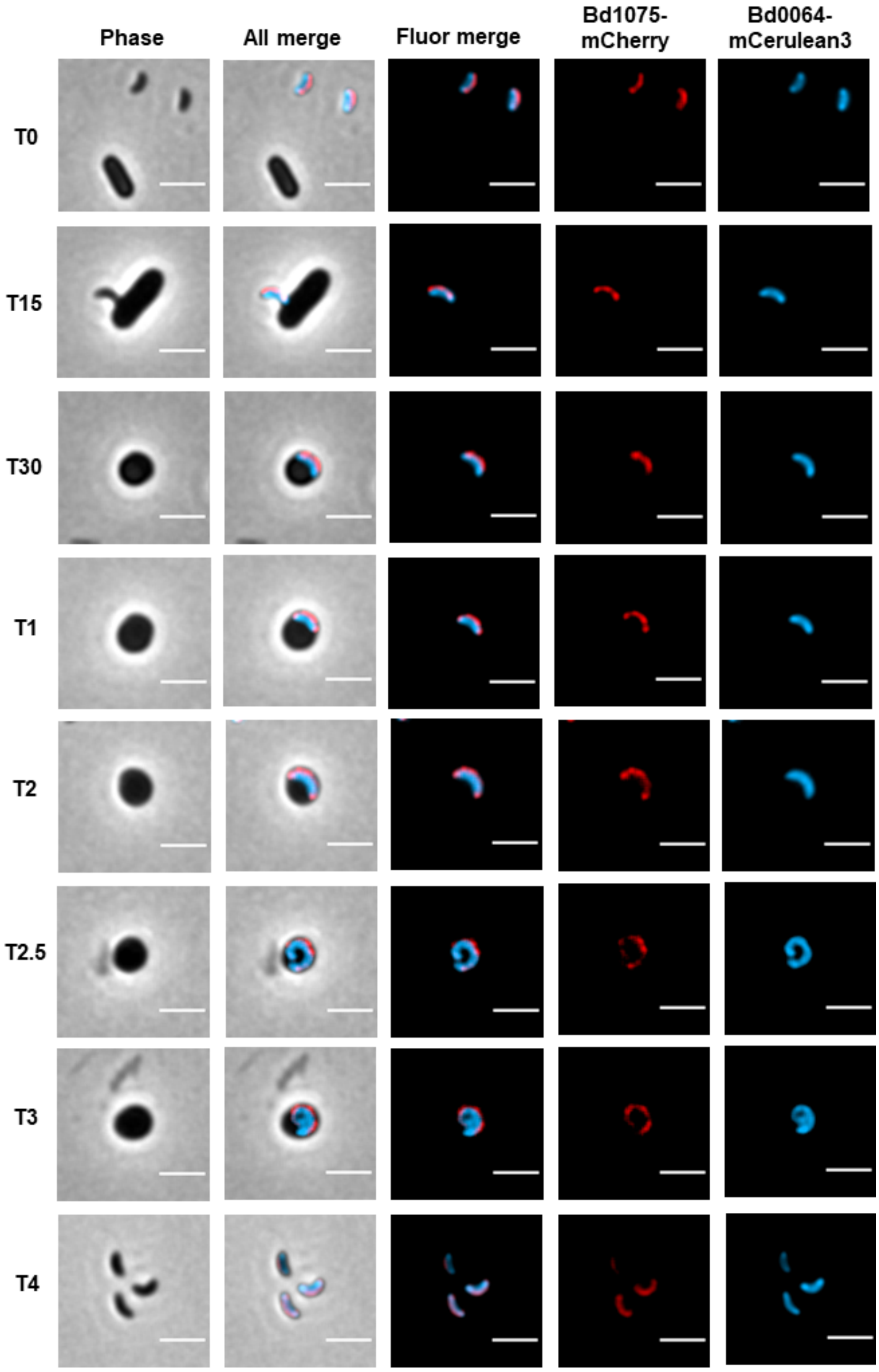

Supplementary Fig. 19 Localization of Bd1075-mCherry during the predatory cycle of *B. bacteriovorus*

Growth of *B. bacteriovorus* HD100 containing chromosomal fusions of both Bd0064-Cerulean3 (to label the predator cytoplasm) and Bd1075-mCherry inside *E. coli* S17-1 pZMR100 prey bdelloplasts. T = min (0-30) or hours (1-4) elapsed since predators and prey were initially mixed. Scale bars = 2 μm. Images are representative of cells from 3 biological repeats.

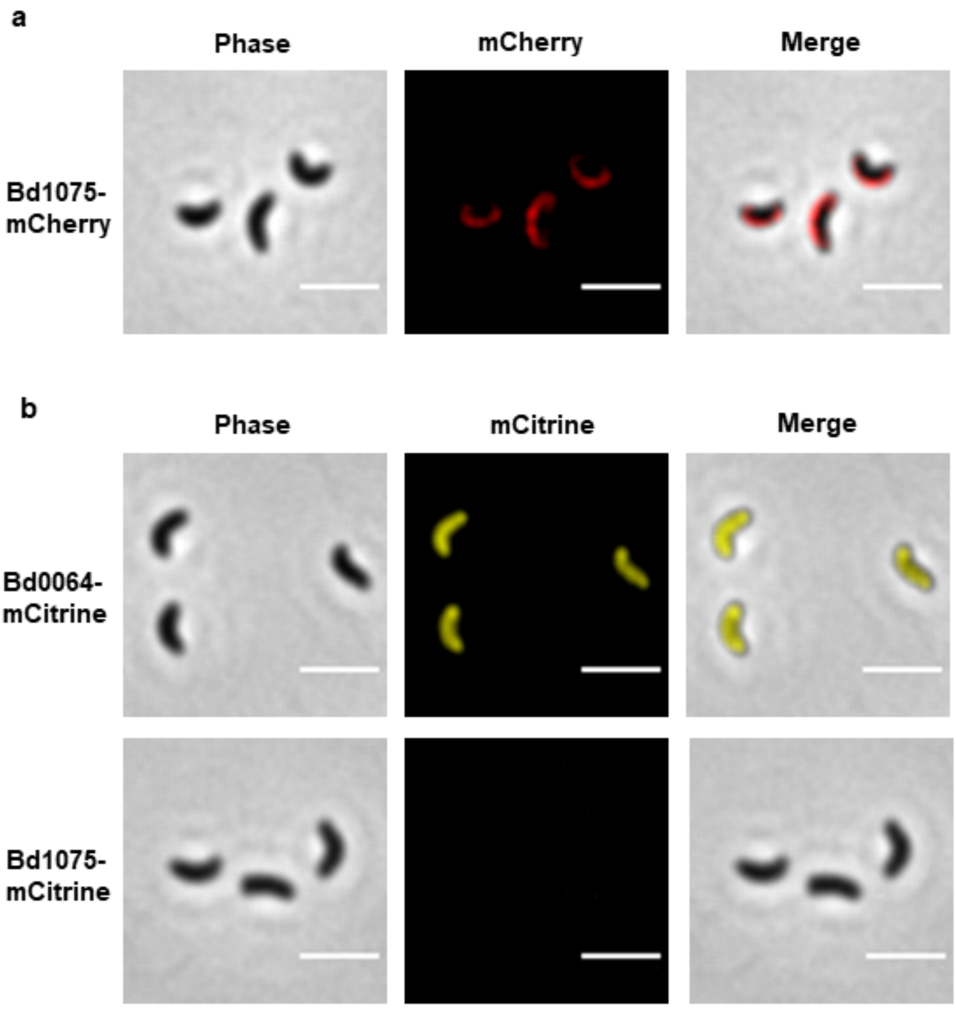

Supplementary Fig. 20 Periplasmic localization of Bd1075

B. bacteriovorus HD100 attack-phase cells containing a single-crossover chromosomal fusion of Bd1075-mCherry (**a**) or a single-crossover chromosomal fusion of either Bd0064 or Bd1075 to the fluorophore mCitrine which, unlike mCherry, cannot fluoresce in the bacterial periplasm (**b**). The cytoplasmic protein Bd0064-mCitrine shows fluorescence but the periplasmic protein Bd1075-mCitrine does not. All strains were sequenced to confirm that each fusion was correctly constructed. Scale bars = 2 μm and images are representative of 3 biological repeats.

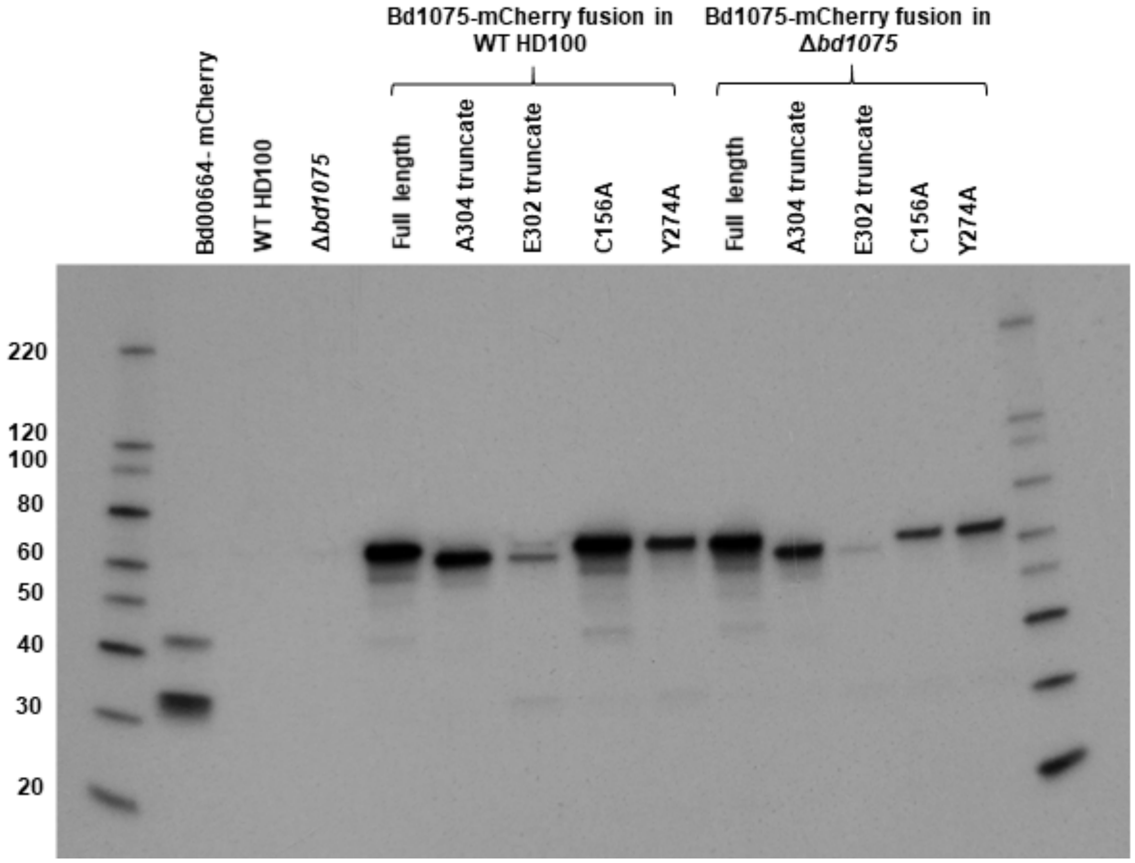

Supplementary Fig. 21 Western blot confirming production of Bd1075-mCherry fusions

Western blot showing the stable production of Bd1075-mCherry fluorescent fusions in attack-phase *B. bacteriovorus* used in Fig. 6. Fusions were detected with an anti-mCherry primary antibody. Bd0064-mCherry is a positive control for mCherry detection and WT HD100 and Δ*bd1075* are negative controls with no fluorescent tags. Full length Bd1075-mCherry, A304 truncate-mCherry, E302 truncate-mCherry, C156A (point mutation)-mCherry and Y274A (point mutation)-mCherry were detected in both a wild-type background and a Δ*bd1075* background at the expected approximate size of 62 kDa (mCherry: 25 kDa + Bd1075: 37 kDa). The E302 truncated variant was present in lower levels, possibly representing a slightly less stable protein, but was present in sufficient quantities for the fluorescence and location to be accurately determined. The western blot is representative of three independent repeats.

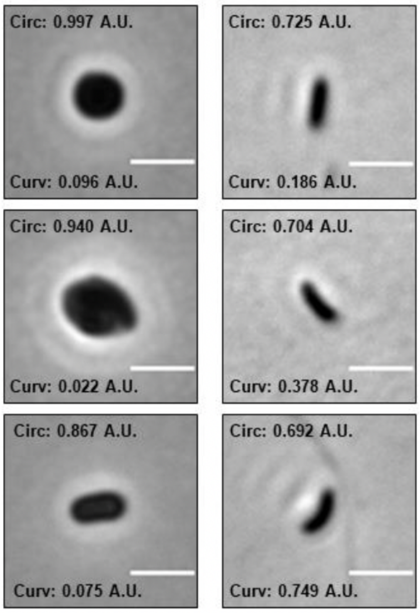

Supplementary Fig. 22 Examples of curvature and circularity measurements by MicrobeJ software

Circularity (Circ) and curvature (Curv) values in arbitrary units (A.U.) are shown above and below each bacterial (*E. coli* prey: left and *B. bacteriovorus* predators: right), respectively. Circularity was used to assess prey bdelloplast shape and curvature to measure *B. bacteriovorus* cell curvature. Scale bars = 2 µm.

Supplementary Table 1. Quantification of muropeptides released from *B. bacteriovorus* HD100 and 109J sacculi.

Values represent the relative percentage area of each muropeptide peak in **Supplementary Fig. 11**. For the strain HD100 Δ*bd1075* (p*bd1075*$_{109J}$), numbers with an asterisk differ from WT HD100 values (Table 1) by more than 30% and numbers that are additionally emboldened differ by more than 50%. Values of strains 109J (p*bd1075*$_{HD100}$) and 109J (pEV) are compared to WT 109J in identical manner. Source Data are provided as a Source Data file.

Muropeptide	Relative peak area (%)[1] in *B. bacteriovorus* strain			
	**Δ*bd1075* (p*bd1075*$_{109J}$)**	**WT 109J**	**109J (p*bd1075*$_{HD100}$)**	**109J (pEV)**
**Monomers**				
Tri	**n.d.*[2]**	n.d.	**18.7 ± 3.0***	0.3 ± 0.4
Tetra	**22.2 ± 1.5***	28.0 ± 4.3	**3.0 ± 0.8***	26.4 ± 0.5
Di	**n.d.***	n.d.	**6.2 ± 0.8***	n.d.
Penta	12.8 ± 5.9	13.1 ± 4.1	14.0 ± 1.9	14.6 ± 2.5
Monomer anhydroMurNAc	2.8 ± 4.0*	2.5 ± 3.5	2.2 ± 3.2	3.1 ± 4.3
Monomers (Total)	35.1 ± 7.4	41.0 ± 8.4	42.0 ± 4.8	41.3 ± 2.7
**Dimers**				
TetraTri	**0.5 ± 0.7***	0.3 ± 0.4	**26.2 ± 0.6***	0.2 ± 0.2*
TetraTetra	**33.4 ± 0.9***	33.8 ± 1.1	**8.8 ± 0.8***	34.3 ± 1.5
TetraPenta	12.6 ± 0.5	11.4 ± 1.4	13.2 ± 0.5	12.1 ± 0.9
Dimer anhydroMurNAc	17.2 ± 1.3	15.1 ± 1.3	16.2 ± 1.1	15.2 ± 0.4
Dimers (total)	46.5 ± 0.6	45.5 ± 0.7	48.2 ± 1.0	46.6 ± 0.3
**Trimers**				
TetraTetraTri	**n.d.***	n.d.	**2.3 ± 0.6***	n.d.
TetraTetraTetra	**11.6 ± 2.6***	7.9 ± 1.9	**2.5 ± 0.4***	7.2 ± 0.9
TetraTetraPenta	6.9 ± 5.4	5.6 ± 5.8	5.1 ± 4.8	4.9 ± 4.0
Trimer anhydroMurNAc	10.4 ± 2.8*	6.6 ± 2.5	**2.9 ± 1.0***	5.9 ± 0.7
Trimers (total)	18.4 ± 8.0	13.5 ± 7.7	9.8 ± 5.8	12.1 ± 3.0
Dipeptides (total)	**n.d.***	n.d.	**6.2 ± 0.8***	n.d.
Tripeptides (total)	**0.2 ± 0.3***	0.1 ± 0.2	**32.6 ± 3.1***	**0.3 ± 0.5***
Tetrapeptides (total)	78.3 ± 4.0*	79.3 ± 1.3	**38.9 ± 2.3***	77.4 ± 0.3
Pentapeptides (total)	18.6 ± 0.3	18.1 ± 2.0	20.0 ± 3.2	19.2 ± 3.6
AnhydroMurNAc (total)	14.9 ± 2.4	12.3 ± 2.1	11.3 ± 3.4	12.6 ± 4.8
Average chain length	6.8 ± 1.1	8.3 ± 1.4	9.2 ± 2.8	8.5 ± 3.2
Degree of cross-linkage	35.5 ± 5.0	31.7 ± 5.5	30.7 ± 3.4	31.4 ± 1.9
% peptides in cross-links	64.9 ± 7.4	59.0 ± 8.4	58.0 ± 4.8	58.7 ± 2.7

[1] values are mean ± variation of two biological replicates.
[2] n.d., not detected.

Supplementary Table 2. Reduced muropeptides from *B. bacteriovorus* wild-type HD100 collected from HPLC and analysed by mass spectrometry.

Peak No.	Muropeptide[1]	Measured neutral mass (Da)	Theoretical neutral mass (Da)
8	TetraTetraTri	2717.90	2717.16
9	TetraTetraTetra	2788.74	2788.20
10	PentaAnh	992.39	992.42
11	TetraTetraPenta	2859.19	2859.24
12	TetraTriAnh I	1773.65	1773.74
13	TetraTriAnh II	1773.57	1773.74
14	TetraTetraAnh I	1844.57	1844.78
15	TetraTetraAnh II	1844.41	1844.78
16	TetraPentaAnh	1915.96	1915.82
17	TetraTetraTetraAnh	2768.44	2768.18
18	TetraTetraPentaAnh I	2839.10	2839.21
19	TetraTetraPentaAnh II	2839.18	2839.21

[1] Nomenclature of muropeptides according to Glauner (1988)[9].

Supplementary Table 3. Bd1075 structure data collection and refinement statistics.

	Bd1075-Br SAD	Bd1075 native
**Data Collection**		
Resolution range (Å)	40.19 - 1.39	43.92 - 1.34
Space group	$P\ 2_1$	$P\ 2_1$
Cell dimensions		
a, b, c (Å)	49.11, 100.39, 62.28	49.189, 100.631, 62.2787
α, β, γ (°)	90, 108, 90	90, 108.07, 90
Total reflections	593918 (14215)	847431 (69189)
Unique reflections	94881 (4170)	123276 (11323)
Completeness (%)	82.47 (36.26)	95.37 (85.34)
Redundancy	6.3 (3.4)	6.9 (6.1)
$\langle I \rangle / \sigma I$	20.58 (0.72)	17.51 (0.49)
R_{meas}	0.04208 (1.62)	0.06161 (2.08)
R_{merge}	0.03875 (1.39)	0.05678 (1.9)
$CC_{1/2}$	1.00 (0.36)	0.999 (0.33)
CC_{anom}	0.3 (0.0)	
**Refinement**		
Maximum resolution (Å)		1.34
Number of reflections used		122860 (10948)
Number of reflections for R_{free}		6092 (535)
R_{work}		18.6
R_{free}		21.2
RMSD		
Bonds (Å)		0.011
Angles (°)		1.02
Number of non-hydrogen atoms		
Protein		4686
Ligand/ion		61
Water		504
B-factors		
Protein		24.22
Ligand/ion		41.63
Water		34.37
Ramachandran favoured (%)		97.57
Ramachandran allowed (%)		2.43
Ramachandran outliers (%)		0.00
Rotamer outliers (%)		0.39

Supplementary Table 4. Primers used in this research study.

Primer	Sequence (5' → 3')	Purpose
1075_up_F	CGTTGTAAAACGACGGCCAGTGCCAGACCATCGGAACCGCGTG	Deletion of *bd1075*
1075_up_R	CTAGTTTATTGCGTCTCATAAATACTATTATGCCCGAATAGGAC	
1075_down_F	AGTATTTATGAGACGCAATAAACTAGGCTGTAAAG	
1075_down_R	GGAAACAGCTATGACCATGATTACGAGCCAAGTTGGTTTTGTATTC	
1075_HD100c_F	CAGCTGGTACCATATGGGAATTCGAACTTTATTTACATTTAAATTACACGG	Cloning *bd1075*$_{\mathrm{HD100}}$ gene for complementation
1075_HD100c_R	CTTCTCTCATCCGCCAAAACAGCCATATCGAGTTGATGAAAAAAGC	
1075_109Jc_F	CAGCTGGTACCATATGGGAATTCGAACTTTATTTACATTTAAATTACACGG	Cloning *bd1075*$_{\mathrm{109J}}$ gene for complementation
1075_109Jc_R	CTTCTCTCATCCGCCAAAACAGCCAGTATCGAGTTGATGAAAAAAG	
Bd0064_F	CGTTGTAAAACGACGGCCAGTGCCAGTGGAGGACACATATACAGTTC	Bd0064-mCerulean3 single-crossover fusion
Bd0064_R	CTTGCTCACCATTCCGACTTTTTTAAAGATCGTG	
mCeru_F	TAAAAAAGTCGGAATGGTGAGCAAGGGCGAG	
mCeru_R	GGAAACAGCTATGACCATGATTACGTTACTTGTACAGCTCGTCCATG	
1075_up_F	CGTTGTAAAACGACGGCCAGTGCCAGACCATCGGAACCGCGTG	Bd1075-mCherry full-length double-crossover fusion
1075_mCh_up_R	CCTTGCTCACCATTTGCGTTTTCTGGGAAGAGG	
1075_mCh_F	CCAGAAAACGCAAATGGTGAGCAAGGGCGAG	
1075_mCh_R	TTTACAGCCTAGTTTACTTGTACAGCTCGTCCATG	
1075_mCh_down_F	GCTGTACAAGTAAACTAGGCTGTAAAGGCAAAAAAAAAG	
1075_down_R	GGAAACAGCTATGACCATGATTACGAGCCAAGTTGGTTTTGTATTC	
1075_up_genF	CGTTGTAAAACGACGGCCAGTGCCAGACCATCGGAACCGCGTG	Bd1075-mCherry truncation and point mutant fusions (single-crossover)
1075_mCh_SXO_genR	GGAAACAGCTATGACCATGATTACGTTACTTGTACAGCTCGTCCATG	
Full_1075_R	CTTGCTCACCATTTGCGTTTTCTGGGAAGAGG	
Full_mCh_F	CCAGAAAACGCAAATGGTGAGCAAGGGCGAG	
E302_1075_R	CTTGCTCACCATTTCTTCTCGGATGATTTTATAAGTGTCAC	
E302_mCh_F	CATCCGAGAAGAAATGGTGAGCAAGGGCGAG	
A304_1075_R	CTTGCTCACCATAGCCCATTCTTCTCGGATG	
A304_mCh_F	AGAAGAATGGGCTATGGTGAGCAAGGGCGAG	
C156A_F	CTCTCGCGGCGCGGTCGTTGTTCGTAACGACG	
C156A_R	CCGCGGGTCAAAGGCACC	
Y274A_F	CCTGCAGCGCGCGGAATCTGACAAAC	
Y274A_R	GTTTTCACCAGCAACTGATC	
RT_1075_F	CAACGATCAGTTGCTGGTG	RT-PCR primers for expression across predatory lifecycle
RT_1075_R	AAGTGTCACCGGACTTGAGG	
RT_dnaK_F	TGAGGACGAGATCAAACGTG	
RT_dnaK_R	AAACCAGGTTGTCGAGGTTG	
RT_1075_GAP_F	TATTTGCGTTGAGTCTGCCG	RT-PCR primers to test 109J transcript truncation
RT_1075_GAP_R	TGGCTCAGACGCTCTTGC	

Supplementary Table 5. Plasmids used in this research study.

Plasmid	Description	Source
pK18*mobsacB*	Suicide vector (kan^R, *lacZ*α, *sacB*) used for crossovers into the *B. bacteriovorus* genome	[10]
pMQBAD	Plasmid capable of replication within *B. bacteriovorus* and used for complementation (tdTomato, gent^R)	This lab. Originally derived from pMQ414[11]
pAKF56	Template for *mCherry* gene	[12]
pmCerulean3-N1	Template for *mCerulean3* gene	Addgene (#54730)
pET mCitrine LIC cloning vector	Template for *mCitrine* gene	Addgene (#29771)
pdelta1075	Upstream and downstream fragments of *bd1075* gene for unmarked gene deletion	This study
pMQ1075_HD100c	*bd1075* from strain HD100 for plasmid complementation	This study
pMQ1075_109Jc	*bd1075* from strain 109J for plasmid complementation	This study
p1075mCh_DXO	Full length Bd1075-mCherry fusion (double-crossover)	This study
p1075mCh_SXO	Full length Bd1075-mCherry fusion (single-crossover)	This study
p1075_comp	*bd1075* from strain HD100 for single-crossover complementation	This study
p1075mCh_E302_SXO	Bd1075-mCherry fusion terminating at Bd1075 residue E302 (single-crossover)	This study
p1075mCh_A304_SXO	Bd1075-mCherry fusion terminating at Bd1075 residue A304 (single-crossover)	This study
p1075mCh_C156A_SXO	Full length Bd1075-mCherry fusion with a point mutation of C156A (single-crossover)	This study
p1075mCh_Y274A_SXO	Full length Bd1075-mCherry fusion with a point mutation of Y274A (single-crossover)	This study
pbd0064-mCeru_SXO	Bd0064-mCerulean3 (single-crossover)	This study
pbd0064-mCit_SXO	Bd0064-mCitrine (single-crossover)	This study
pbd1075-mCit_SXO	Bd1075-mCitrine (single-crossover)	This study

Supplementary Table 6. Strains used in this research study.

Strain	Description	Source
E. coli DH5α	*E. coli* cloning strain (F- endA1 hsdR17 (rk -mk -) supE44 thi-1 recA1 gyrA (Nalr) relA1 Δ(lacIZYA-argF) U169 deoR (80dlacΔ (lacZ)M15))	[13]
E. coli S17-1	*E. coli* strain (thi, pro, hsdR-, hsdM+, recA; integrated plasmid RP4- Tc::Mu-Kn::tn)	[14]
E. coli S17-1: pZMR100	*E. coli* strain containing the plasmid pZMR100 (kan^R)	[15]
E. coli S17-1: pUC19	*E. coli* strain containing the plasmid pUC19 (gent^R)	This lab
E. coli S17-1: lux	*E. coli* S17-1 strain (luminescent)	Gift from Dr Philip Hill
B. bacteriovorus HD100	*B. bacteriovorus* Type strain, genome-sequenced, wild-type	[16]
B. bacteriovorus 109J	*B. bacteriovorus* strain, genome-sequenced, wild-type	This lab
B. bacteriovorus HD100 Δ*bd1075*	*B. bacteriovorus* containing an in-frame unmarked deletion of *bd1075*	This study
B. bacteriovorus HD100 Δ*bd1075:* p1075_HD100c	HD100 Δ*bd1075* containing the complementation plasmid p1075_HD100c	This study
B. bacteriovorus HD100 Δ*bd1075*: p1075_109Jc	HD100 Δ*bd1075* containing the complementation plasmid p1075_109Jc	This study
B. bacteriovorus HD100 Δ*bd1075*: pMQBAD	HD100 Δ*bd1075* containing the empty complementation plasmid	This study
B. bacteriovorus 109J: p1075_HD100c	109J containing the complementation plasmid p1075_HD100c	This study
B. bacteriovorus 109J: pMQBAD	109J containing the empty complementation plasmid	This study
B. bacteriovorus HD100 Bd1075mCh_DXO	HD100 containing a double-crossover, full length Bd1075-mCherry fusion	This study
B. bacteriovorus HD100 Bd1075mCh_SXO	HD100 containing a single-crossover, full length Bd1075-mCherry fusion	This study
B. bacteriovorus HD100 Bd1075mCh_DXO_64mCerulean3_SXO	HD100 containing both a double-crossover, full length Bd1075-mCherry fusion and a single-crossover Bd0064-mCerulean3 fusion.	This study
B. bacteriovorus HD100 Bd1075mCh_E302_SXO	HD100 containing a single-crossover Bd1075-mCherry fusion terminating at Bd1075 residue E302	This study
B. bacteriovorus HD100 Bd1075mCh_A304_SXO	HD100 containing a single-crossover Bd1075-mCherry fusion terminating at Bd1075 residue A304	This study
B. bacteriovorus HD100 Bd1075mCh_C156A_SXO	HD100 containing a single-crossover, full length Bd1075-mCherry fusion with the point mutation C156A	This study
B. bacteriovorus HD100 Bd1075mCh_Y274A_SXO	HD100 containing a single-crossover, full length Bd1075-mCherry fusion with the point mutation Y274A	This study
B. bacteriovorus HD100 Δ*bd1075* Bd1075mCh_SXO	HD100 Δ*bd1075* containing a single-crossover, full length Bd1075-mCherry fusion	This study
B. bacteriovorus HD100 Δ*bd1075* Bd1075mCh_E302_SXO	HD100 Δ*bd1075* containing a single-crossover Bd1075-mCherry fusion terminating at Bd1075 residue E302	This study

B. bacteriovorus HD100 Δ*bd1075* Bd1075mCh_A304_SXO	HD100 Δ*bd1075* containing a single-crossover Bd1075-mCherry fusion terminating at Bd1075 residue A304	This study
B. bacteriovorus HD100 Δ*bd1075* Bd1075mCh_C156A_SXO	HD100 Δ*bd1075* containing a single-crossover, full length Bd1075-mCherry fusion with the point mutation C156A	This study
B. bacteriovorus HD100 Δ*bd1075* Bd1075mCh_Y274A_SXO	HD100 Δ*bd1075* containing a single-crossover, full length Bd1075-mCherry fusion with the point mutation Y274A	This study
B. bacteriovorus HD100 Bd0064mCeru_SXO	HD100 containing a single-crossover Bd0064-mCerulean3 fusion	This study
B. bacteriovorus HD100 Δ*bd1075* Bd0064mCeru_SXO	HD100 Δ*bd1075* containing a single-crossover Bd0064-mCerulean3 fusion	This study
B. bacteriovorus HD100 Δ*bd1075* (*bd1075*comp)	HD100 Δ*bd1075* containing a single-crossover complementing copy of *bd1075*.	This study
B. bacteriovorus HD100 Bd0064mCit_SXO	HD100 containing a single-crossover Bd0064-mCitrine fusion	This study
B. bacteriovorus HD100 Bd1075mCit_SXO	HD100 containing a single-crossover Bd1075-mCitrine fusion	This study

Supplementary references

1. Chaudhuri RR, Pallen MJ. xBASE, a collection of online databases for bacterial comparative genomics. *Nucleic Acids Res.* **34**, D335-337 (2006).

2. Capeness MJ*, et al.* Activity of *Bdellovibrio* hit locus proteins, Bd0108 and Bd0109, links type IVa pilus extrusion/retraction status to prey-independent growth signalling. *PLoS One* **8**, e79759 (2013).

3. McClure R*, et al.* Computational analysis of bacterial RNA-Seq data. *Nucleic Acids Res.* **41**, e140 (2013).

4. Tjaden B. De novo assembly of bacterial transcriptomes from RNA-seq data. *Genome Biol.* **16**, 1 (2015).

5. Robinson JT*, et al.* Integrative genomics viewer. *Nat. Biotechnol.* **29**, 24-26 (2011).

6. Madeira F*, et al.* The EMBL-EBI search and sequence analysis tools APIs in 2019. *Nucleic Acids Res.* **47**, W636-W641 (2019).

7. Robert X, Gouet P. Deciphering key features in protein structures with the new ENDscript server. *Nucleic Acids Res.* **42**, W320-324 (2014).

8. Wurtzel O, Dori-Bachash M, Pietrokovski S, Jurkevitch E, Sorek R. Mutation detection with next-generation resequencing through a mediator genome. *PLoS One* **5**, e15628 (2010).

9. Glauner B. Separation and quantification of muropeptides with high-performance liquid chromatography. *Anal. Biochem.* **172**, 451-464 (1988).

10. Schafer A, Tauch A, Jager W, Kalinowski J, Thierbach G, Puhler A. Small mobilizable multi-purpose cloning vectors derived from the *Escherichia coli* plasmids pK18 and pK19: selection of defined deletions in the chromosome of *Corynebacterium glutamicum*. *Gene* **145**, 69-73 (1994).

11. Mukherjee S, Brothers KM, Shanks RMQ, Kadouri DE. Visualizing *Bdellovibrio bacteriovorus* by using the tdTomato fluorescent protein. *Appl Environ Microbiol* **82**, 1653-1661 (2015).

12. Fenton AK, Kanna M, Woods RD, Aizawa SI, Sockett RE. Shadowing the actions of a predator: backlit fluorescent microscopy reveals synchronous nonbinary septation of predatory *Bdellovibrio* inside prey and exit through discrete bdelloplast pores. *J. Bacteriol.* **192**, 6329-6335 (2010).

13. Simon R, Priefer U, Pühler A. A broad host range mobilization system for *in vivo* genetic engineering: transposon mutagenesis in Gram negative bacteria. *Biotechnology. (N. Y.)* **1**, 784-791 (1983).

14. Hanahan D. Studies on transformation of *Escherichia coli* with plasmids. *J. Mol. Biol.* **166**, 557-580 (1983).

15. Rogers M, Ekaterinaki N, Nimmo E, Sherratt D. Analysis of Tn7 transposition. *Mol Gen Genet* **205**, 550-556 (1986).

16. Rendulic S*, et al.* A predator unmasked: life cycle of *Bdellovibrio bacteriovorus* from a genomic perspective. *Science* **303**, 689-692 (2004).
