# Supplementary material for: Asymmetric peptidoglycan editing generates cell curvature in Bdellovibrio predatory bacteria
Source: Nat Commun. 2022 Mar 21;13:1509. doi: 10.1038/s41467-022-29007-y (PMC8938487; doi:10.1038/s41467-022-29007-y)
Supplement: Supplementary file 4 — Description of Additional Supplementary Files [file 41467_2022_29007_MOESM4_ESM.pdf]

Description of additional supplementary files

File name: Supplementary Movie 1:

Description**: *B. bacteriovorus* HD100 wild-type invasion into *E. coli* prey.**

The time interval between frames was 1 min and the scale bar = 2 µm. The corresponding image series is shown in Supplementary Fig. 11.

File name: Supplementary Movie 2:

Description: ***B. bacteriovorus* HD100 Δ*bd1075* invasion into *E. coli* prey.**

The time interval between frames was 1 min and the scale bar = 2 µm. The corresponding image series is shown in Supplementary Fig. 11.

File name: Supplementary Movie 3:

Description: ***B. bacteriovorus* HD100 Δ*bd1075* (comp) invasion into *E. coli* prey.**

The time interval between frames was 1 min and the scale bar = 2 µm. The corresponding image series is shown in Supplementary Fig. 11.
